# Heart failure and excess mortality after aortic valve replacement in aortic stenosis

**DOI:** 10.1080/14779072.2023.2186853

**Published:** 2023-03-29

**Authors:** Nikoo Aziminia, Christian Nitsche, Rok Mravljak, Jonathan Bennett, George D Thornton, Thomas A Treibel

**Affiliations:** a Institute of Cardiovascular Science, University College London, London, England; b Barts Heart Centre, London, England

**Keywords:** Aortic stenosis, aortic valve replacement, heart failure, myocardial fibrosis, left ventricular hypertrophy

## Abstract

**Introduction:**

In aortic stenosis (AS), the heart transitions from adaptive compensation to an AS cardiomyopathy and eventually leads to decompensation with heart failure. Better understanding of the underpinning pathophysiological mechanisms is required in order to inform strategies to prevent decompensation.

**Areas covered:**

In this review, we therefore aim to appraise the current pathophysiological understanding of adaptive and maladaptive processes in AS, appraise potential avenues of adjunctive therapy before or after AVR and highlight areas of further research in the management of heart failure post AVR.

**Expert opinion:**

Tailored strategies for the timing of intervention accounting for individual patient’s response to the afterload insult are underway, and promise to guide better management in the future. Further clinical trials of adjunctive pharmacological and device therapy to either cardioprotect prior to intervention or promote reverse remodeling and recovery after intervention are needed to mitigate the risk of heart failure and excess mortality.

## 1. Introduction

Valvular heart disease (VHD) presents a significant disease burden globally, predominantly of functional and degenerative etiology in high-income countries and rheumatic etiology in middle- and low-income countries. The true global burden remains unknown in the absence of universal availability of cardiovascular imaging [1,2]. Aortic stenosis (AS) is the most common VHD in the western world; >400,000 people underwent aortic valve replacement (AVR) in the USA alone in 2019 [3]. Current guidelines recommend AVR to improve survival in the context of severe AS where symptoms emerge, either spontaneously or on exercise testing, or there is a reduction in left ventricular (LV) function [4]. Years of excessive afterload can result in an ‘AS cardiomyopathy’ with concentric LV hypertrophy (LVH), remodeling of myocytes and the extracellular matrix, and capillary rarefaction [5]. This culminates in ischemia, and diffuse and focal fibrosis. Adverse remodeling, in particular myocardial scar, are drivers of adverse outcomes even after successful AVR, leading to poor prognosis [6,7].

This excess risk has been poorly captured in the literature, with lack of evidence about the mechanisms of excess mortality which could guide potential treatment options. As a result, current VHD guidelines offer limited guidance on the management of valvular heart failure after valve intervention.

Identification of pathological mechanisms underlying heart failure post AVR and appropriate therapeutic strategies post intervention may enhance our precision treatment of patients referred for AVR and improve general AS management beyond valve replacement. In this review, we therefore aim to appraise the current evidence of heart failure mechanisms in AS (before and after AVR) and highlight areas of further research in the management of heart failure post-AVR.

### 1.1. Natural history of aortic stenosis – valve and ventricle

The natural history of AS is largely determined by the interplay between the stenotic valve, the myocardium, and the vasculature, where the response of the LV ultimately determines symptoms and outcomes. AS has historically been attributed to age-related ‘wear and tear,’ however its pathogenesis occurs as a result of complex processes similar to those occurring in atherosclerosis. The initiating process is thought to be endothelial damage secondary to mechanical wall stress and reduced shear stress, which combined with lipid deposition can trigger valvular inflammation. Later pathology is dominated by valvular fibrosis, calcification, and neo-angiogenesis within close proximity of areas of inflammation [8].

The stages of AS can be defined according to the anatomy of the valve, the presence of symptoms, valve hemodynamics and the consequences of these for the structure and function of the LV. A patient with normal valve hemodynamics at risk of developing AS (Stage A) progresses to valvular obstruction with hemodynamic changes (Stage B), with no symptoms at this stage. This may develop into asymptomatic severe AS (Stage C, divided according to the absence or presence of LV systolic dysfunction at Stages C1 and C2 respectively). Ultimately, the patient may experience symptoms (Stage D), which can be further subdivided based on the hemodynamic profile: high gradient (D1), low-gradient and low-flow with impaired LV systolic function (D2), or with low-gradient and low-flow but preserved systolic function or paradoxical low-flow (D3) [9]. Genereux et al. developed a staging system according to the degree of extra-valvular cardiac damage, according to the presence of LV, LA or mitral valve, pulmonary vasculature or tricuspid valve, and RV damage. The trend according to this stratification model demonstrated a significant association with all-cause mortality, cardiovascular mortality, and a composite of death, hospitalization or stroke [10].

The pressure overload due to the stenotic aortic valve triggers a hypertrophic response within the LV myocardium. The pattern of remodeling can vary between individuals, under the influence of factors including sex, age, the presence of co-existent coronary artery disease (CAD) or hypertension [11–13]. AS progression is also associated with changes in coronary microvascular function. Coronary blood flow is balanced between intravascular coronary arterial pressure and extravascular tissue pressure [14,15]. With increasing afterload of AS and thereby pressure, LV remodeling and hypertrophy may lead to increased wall stress. While a compensatory mechanism, this is not without drawbacks. Reduced coronary vasodilatory reserve is demonstrable in patients with AS and LVH as well as reduced diastolic perfusion time, a key mechanism of myocardial ischemia in AS [16]. Together with the raised myocardial oxygen demand in progressive AS, this leads to a supply-demand mismatch which may result in anginal symptoms [15]. The effects of such pathological myocardial remodeling in the context of AS may be compounded by underlying coronary artery disease, a multi-faceted problem which may be addressed by concurrent coronary revascularisation along with AVR, improving coronary hemodynamics as well as valve hemodynamics [17]. Myocardial fibrosis is thought to occur through apoptosis of hypertrophied cardiomyocytes with subsequent replacement by fibrotic tissue [7,6]. The presence of fibrosis is associated with the progression from LVH to heart failure, arrhythmias, and increased risk of sudden cardiac death [6,7,18]. Evidence of fibrosis on cardiovascular magnetic resonance (CMR) is independently associated with increased mortality in patients with AS [18].

### 1.2. Current management of AS

Our understanding of AS-related mortality and the technology to treat it have dramatically changed over the last 50 years. First, the availability of surgical AVR (SAVR) and transcatheter aortic valve implantation (TAVI) has transformed outcomes for patients with AS. Improvement of surgical techniques has led to a reduction in mortality risk associated with isolated SAVR, now quoted to be as low as 1% [19]. Recent randomized clinical trials indicate non inferiority of TAVI over AVR on moderate and low-risk patients [20,21,22]. Second, the demographics of our AS patients have changed to include elderly degenerative AS and not just the younger bicuspid patients described by Braunwald and Ross. Third, watchful waiting fails in a significant percentage of patients. In one study, 61% of patients presenting with decompensated AS were in watchful waiting and their in-hospital mortality was a staggering 16% [23]. Finally, adverse cardiac remodeling starts in patients with moderate valve stenosis and already results in potentially irreversible damage.

Despite advances in aortic valve procedures, outcomes remain poor. Timing of intervention is critical to mitigate irreversible damage. There is trial evidence that intervention in asymptomatic patients with severe or very severe AS translates into reduced mortality, regression of LVH and sustained LV systolic function, suggestive of reverse remodeling prior to irreversible myocardial damage [24]. Randomized controlled trials (RCT; AVATAR and RECOVERY) of early surgical intervention in asymptomatic severe AS have recently reported clear mortality benefits of early AVR versus current guideline based management, but patients in these studies had very low operative risk, were young [25] and had very high gradients [19]. To be applicable to a broader range of AS patients with multiple comorbidities at moderate to high interventional risk, we need further RCT data. Pragmatic RCTs of early intervention versus watchful waiting are under way with the EASY-AS and EARLY TAVR trials [26,27]. New strategies of intervention need to integrate these changes (patient demographics, understanding of the impact of cardiac damage, improvements in our techniques) and balance them with the upfront risks of early intervention (complications of SAVR/TAVI, living with a prosthetic valve, anticoagulation, repeat intervention for structural valve deterioration) as part of an individually tailored management strategy for patients with AS.

### 1.3. Prevalence of heart failure pre- and post-AVR

Presentation with heart failure prior to AVR is common, and is an overlooked and life- threatening problem. Wald et al showed that out of 684 patient admissions coded with a diagnosis of aortic stenosis, 141 (21%) emergencies admission with decompensation of AS and these patients had a high in-hospital mortality of 16% [23]. Across recent studies of surgical and transcatheter AVR, the prevalence of impaired left ventricular ejection fraction prior to AVR ranges between 10% and 15% for LVEF<30% and as high as 35% for LVEF<40% – a quarter of patients in the PARTNER trials had an LVEF between 20% and 50%.

Although AVR significantly attenuates the natural history of AS and reduces subsequent progression to heart failure and death [28], it is not surprising that patients with prior heart failure are more likely to present with heart failure even after successful AVR.

Post TAVI, 11.7% of patients experience a readmission due to a cardiovascular cause at 1 year, with heart failure the most common cause, and being associated with increased mortality [29]. In the FRANCE-2 registry of 5-year outcome after TAVI with 4201 patients were enrolled in 34 centers. Heart failure rate in 1 year was 14% and then about 5% in the subsequent years [30]. Similar data was shown in the analysis of risk factors for excess mortality after aortic valve replacement [31]. These studies and registries clearly show that there is still a significant incidence and prevalence of cardiac decompensation and cardiovascular mortality after AVR. In the following sections, we will explore the etiologies of cardiac decompensation.

## 2. Etiology for post AVR heart failure

### 2.1. Peri- and post-operative complications and prosthesis selection

**Figure 1.**
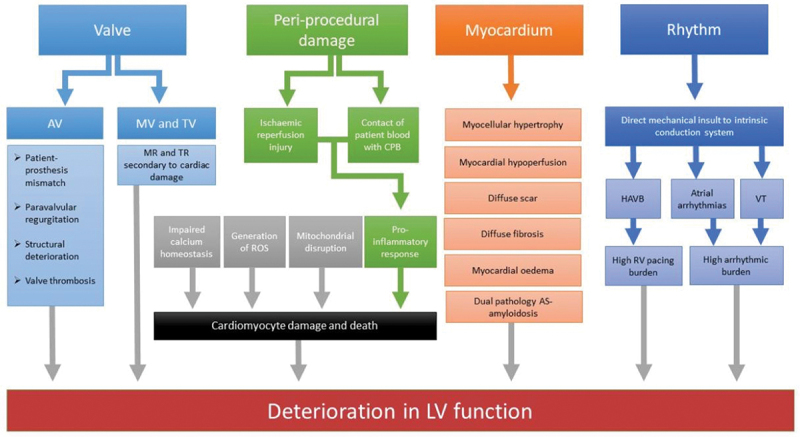
Contributors to heart failure after aortic valve replacement. AS = aortic stenosis, AV = aortic valve, MV = mitral valve, TV = tricuspid valve, ROS = reactive oxygen species, CPB = cardiopulmonary bypass, HAVB = higher degree atrioventricular block, LV = left ventricle, RV = right ventricle, VT = ventricular tachycardia

Although there have been significant advances in the implantation techniques and longevity of prosthetic valves, complications affecting their structure and hemodynamic performance persist. These may be evaluated as structural valve dysfunction, due to problems inherent in the prosthesis, or non-structural valve dysfunction, in the absence of these using a defined staging system as proposed by Pibarot and colleagues [32]. Exploring prosthetic valve dysfunction and the resultant persistently elevated hemodynamic load and LVH following valve replacement, and addressing these, may serve to improve outcomes following AVR (Figure 1).

#### 2.1.1. Patient-prosthesis mismatch

Patient prosthesis mismatch (PPM) occurs where the effective orifice area of the prosthetic valve is too small relative to the patient’s body habitus, resulting in high transvalvular gradients. The incidence of PPM is different according to the type of intervention and the bioprosthetic model used. A higher prevalence and severity of PPM has been demonstrated for SAVR compared to TAVI; the PARTNER trial investigators demonstrated an incidence of 46.4% in patients following TAVI compared with 60% following SAVR [33]. However, there is variability among studies. In a meta-analysis on PPM after SAVR the incidence of moderate/severe PPM was 54%, ranging from 6% to 94% according to different reports [34]. Conversely, the PPM incidence after TAVI was only 24% according to a recent meta-analysis [35].

Given these potentially detrimental effects of PPM, clinicians efforts should be maximized to avoid PPM. Re-intervention may be indicated if the patient develops symptoms attributable to PPM or if unfavorable hemodynamic effects develop, with the options of re-do surgery or a transcatheter valve-in-valve (ViV) procedure, with fracturing of the surgical valve stent [36]. As demonstrated by data from the VIVID registry, preexisting severe PPM confers worse prognosis following ViV, highlighting the importance of preventing it in the first place [37].

#### 2.1.2. Paravalvular regurgitation

Another important hemodynamic sequela after AVR is paravalvular regurgitation (PVR), which can contribute to the residual hemodynamic load of the LV and can also be associated with reduced regression of LVH. PVR can occur due to undersizing, malpositioning, or lack of adequate sealing due to irregularities or calcification of the native AV annulus. While PPM is more frequent after SAVR, significant PVR is observed predominantly following TAVI rather than SAVR [38]. There is variability in the observed frequency of PVR among different TAVI models, underscoring the importance of thorough pre-procedural planning to guide optimal prosthesis selection and positioning for valve deployment [39,40]. Both paravalvular and total aortic regurgitation following any form of AVR are associated with increased mortality, with the effect on mortality proportional to the severity of regurgitation [41]. Following TAVI, PVR of moderate or higher degree is a predictor of worse outcome [30]. If PVR is recognized during the TAVI procedure, post-dilatation may be effective in the reduction of PVR. Post-dilatation as a separate, stand-alone procedure may improve the degree of PVR and transvalvular gradients, as well as symptoms [42]. The development of percutaneous paravalvular leak closure devices may in the future yield a valuable treatment option to avoid valve-in-valve procedures in patients with significant PVR and hemodynamic sequelae.

#### 2.1.3. Structural valve deterioration

Structural deterioration is the major determinant of bioprosthetic longevity. Serial echocardiographic assessment is the principal modality to assess longitudinal hemodynamic changes of the prosthetic performance and establish a diagnosis of structural valve deterioration (SVD). SVD rates of surgical aortic bioprostheses from real-world data are in the range of 4% to 5% per valve-year [43,44]. 5-year data from the PARTNER-2 trial suggest lower durability of the second generation and equal durability of the third-generation balloon-expandable valve when compared to SAVR [39]. Conversely, the NOTION trial found a statistically significant difference in SVD at 8-year follow-up between TAVI and SAVR favoring the former [45]. The CoreValve investigators also found a lower rate of moderate SVD following TAVI compared with SAVR over a 5-year follow-up period, although there was no significant difference between the two groups for rates of severe SVD [46]. Further data on long-term hemodynamic performance of TAVI prostheses is being collected. The occurrence of SVD after SAVR is associated with increased morbidity and mortality, which highlights the necessity of improved valve design and close clinical follow-up of affected patients to assess optimal timing of re-intervention [43,44,47,48].

#### 2.1.4. Valve thrombosis

Valve thrombosis is a recognized structural complication following both TAVI and SAVR. The risk of valve thrombosis in mechanical valves is largely mitigated by therapeutic oral anticoagulation. Bioprosthetic valve material is also thrombogenic, with the risk of thrombosis remaining highest within 3 months of implantation until endothelialisation of the stent material occurs. Nevertheless, the risk of thrombosis of bioprosthetic aortic valves remains low, with a rate of 3% per 100 patient years according to a large meta-analysis [49]. Valve thrombosis can occur following TAVI with multidetector computed tomography identifying hypo-attenuated leaflet thickening (HALT) in ~10% of patients [50,51]. However, HALT does not have to be accompanied by abnormal valve hemodynamics or symptoms [51]. Patients with symptomatic clinical valve thrombosis (thromboembolism and/or elevated transvalvular gradients) have an increased risk of stroke and hemodynamic compromise, and should therefore be treated. Management options include anticoagulation, surgery, or thrombolysis [52]. Asymptomatic valve thrombosis/HALT was not found to be associated with mortality or stroke at 3 years after TAVI, and therefore does not require specific treatment according to current recommendations [53,54]. There is limited data to inform management of bioprosthetic valvular thrombosis, although a prospective study in patients with suspected thrombosis treated with warfarin demonstrated reduction in valve gradient [55]. In the event of hemodynamic instability or failure to respond to anticoagulant therapy, surgery and fibrinolysis may be considered similarly to mechanical valvular thrombosis.

### 2.2. Peri-procedural damage

Myocardial stressors during cardiac surgery such as ischemia, ischemia-reperfusion injury, cardioplegia, inflammation and myocyte necrosis can influence the post-operative course in the short term and prognosis in the long term. It is well recognized that cardiac surgery and cardiopulmonary bypass (CPB) present a significant physiological and immunological injury to the body. Ischemia-reperfusion injury following CPB can induce a pro-inflammatory response, both secondary to contact of patient blood with the CPB tube system as well as to ischemic-perfusion injury [56]. Pro-inflammatory cytokines such as tumor necrosis factor alpha (TNF-α), interleukin (IL-)1 and IL-6, in addition to soluble substances including Fas, Fas ligand, endotoxin and elastase, have been isolated from patient serum following CPB [57,58,59]. Cardioprotection interventions have largely been investigated in the context of coronary revascularisation and their use has been extended to patients undergoing valve surgery [60]; however, the implicit assumption that metabolic activity is the same in LV myocardium that has undergone remodeling and hypertrophy in the context of AS and non-hypertrophied LV myocardium is arguably flawed [61].

### 2.3. Resultant cardiac damage due to chronic aortic stenosis

AVR decision making mainly relies upon the presence of symptoms attributable to AS in association with echocardiographic criteria of severe AS. Other than reduced LVEF, there are no recommendations with regard to anatomic or functional consequences of AS on the myocardium, despite evidence of the prognostic significance of cardiac damage. As aforementioned, a staging system has been developed according to the extent of cardiac damage and has been correlated with worse outcomes post AVR [10]. Biomarkers such a NTproBNP, troponin, and galectin-3 detected in high levels have been associated with increased all-cause mortality in AS and may portend decompensation. Refining cardiac biomarker panels may present a cheap, accessible and noninvasive means of prognostication of AS [62]. The integration of such a staging system with existing metrics to form a multi-parameter assessment of AS severity and outcome post AVR would further refine the AVR decision making process, ensuring the right patients undergo AVR at the right time.

### 2.4. The myocardium

As discussed above, AS is not solely a disease of the valve but also of the myocardium. The response of the myocardium to the increased hemodynamic load presented by the stenotic valve is a significant determinant of outcome and early identification may therefore potentially guide timely valvular intervention. Severe AS is associated with alterations in myocyte architecture, accumulation of interstitial myocardial fibrosis and edema, and may co-exist with infiltrative myocardial disease.

#### 2.4.1. Myocyte hypertrophy

LVH in AS develops in order to reduce wall stress and maintain cardiac output. Increase in myocellular volume is accompanied by intracellular changes such as modification of intracellular proteins (e.g. titin isoform switch, titin hypophosphorylation). Despite LVH representing a hallmark of LV adaptation to AS, there is considerable heterogeneity in the degree of hypertrophy for a given stenosis severity. Reported factors affecting the magnitude of LVH in AS include presence of arterial hypertension, age, early decline of glomerular filtration rate, metabolic syndrome and obesity, and angiotensin-converting enzyme polymorphism [63–66,67].

#### 2.4.2. Myocardial blood supply

With progressive stenosis, microvascular dysfunction and reduced capillary density ensue, accompanied by impaired myocardial blood flow, diminished coronary reserve, compensatory vasodilation of the remaining vessels, and anginal symptoms. Following observations from CMR-biopsy studies which showed an endo- to epicardial gradient for focal fibrosis, microvascular ischemia is hypothesized to be the main driver of replacement fibrosis in AS [68–70]. Whether impaired myocardial perfusion in severe AS recovers following valve replacement is currently being tested in a large cohort study [71].

#### 2.4.3. Diffuse fibrosis

With progressive LVH, changes in collagen quality and quantity results in diffuse interstitial matrix expansion or diffuse fibrosis. Diffuse fibrosis is assessed via CMR pre- and post-Gadolinium contrast T1 mapping to calculate the extracellular volume fraction (ECV). ECV has been extensively validated against histological fibrosis [72-75]. Outcome studies have established ECV as a powerful independent predictor of mortality and heart failure after valve replacement [7,76].

Paradoxically, at one-year after successful AVR, CMR-ECV was found to increase when compared to preprocedural assessment due to a higher reduction in myocellular volume relative to extracellular space [77]. Novel promising technologies enable ECV derivation from computed tomography (CT) acquisitions, and have also been linked to worse prognosis following valve replacement [78].

#### 2.4.4. Focal scar

Eventually, hypertrophy and resultant microvascular ischemia results in apoptosis of hypertrophied cardiomyocytes with subsequent replacement by fibrotic tissue [6]. Identified nearly 40 years ago in histological and autopsy studies, focal scar can now be identified by CMR late gadolinium enhancement (LGE) imaging, which is considered the gold standard for focal scar assessment. Gadolinium selectively enters the extracellular space, thereby marking areas of increased focal extracellular expansion. Patterns of LGE which can be present in AS range from typical subendocardial infarct LGE, to linear non-infarct, to patchy focal LGE. Overall, focal scar is very common, affecting >50% of elderly, medium-to-high risk AS patients, and is associated with increased hazard of death and cardiovascular events – irrespective of the underlying LGE pattern [79]. Moreover, the greater the scar burden, the higher the mortality [80]. Follow-up CMR data after valve replacement have demonstrated that focal fibrosis is irreversible. These observations have led to the theory that early intervention based on the presence of focal fibrosis rather than AS-related symptoms may have the potential to improve prognosis in severe AS – a hypothesis that is currently being tested in a large randomized controlled trial [81].

#### 2.4.5. Myocardial edema

Recent AS studies have proposed a potential role of oedematous/inflammatory processes in myocardial remodeling using CMR T2 mapping, a technique that is well established in the assessment of myocardial edema [76,82,83]. This aspect might have been overlooked in earlier studies, as ‘free’ myocardial water cannot be detected by myocardial biopsy, but can be detected by T1 and T2 mapping. Data in this area is awaited.

Cardiac decompensation resulting in peripheral edema is a frequent finding in the advanced stages of AS and has been shown to also lead to myocardial edema, thereby also affecting CMR-ECV measurements [76]. Moreover, higher degrees of fluid overload – as determined by quantitative assessment – are associated with progressively worse post-interventional outcomes [76,84].

#### 2.4.6. Dual pathology AS-amyloidosis

Expansion of extracellular space in AS may in some patients encompass mechanisms beyond fibrosis and edema. Cardiac amyloidosis (CA) is an infiltrative disorder which involves the myocardial deposition of misfolded proteins – with transthyretin (ATTR) CA as the most common CA subtype identified in elderly AS patients. As a result of active screening ascertainments, a considerable overlap of severe AS and CA has been found – affecting approximately 1 in 8 patients undergoing TAVI [85,86].

Despite the double hit of AS and CA, no mid-term survival difference after TAVI has been demonstrated for AS-ATTR compared to lone AS [85,86]. However, reverse remodeling differs from AS-ATTR to lone AS, with the former being transferred into a ‘lone ATTR cardiomyopathy’ phenotype by biomarkers, symptoms, and contractility pattern [87]. These differences in reverse remodeling following afterload removal likely contribute to higher rates of heart failure hospitalizations in AS-ATTR versus lone AS [88]. Identification of dual AS-ATTR is important, as novel amyloid-specific treatments are now available with the potential to further improve patient outcomes on top of valvular replacement.

## 3. Reverse remodeling after AVR

While the hemodynamic effects of AVR on the stenotic valve are immediate, the prognosis of AS is largely determined by the ventricular response to relief of outflow obstruction. Regression of remodeling following AVR can predict outcomes [89,90]. This has been studied with both echocardiography and CMR (Table 1). A multi-modality approach can be adopted to assess for reverse remodeling according to a range of parameters assessing anatomy, function, and hemodynamics.

**Table 1.** Left ventricular hypertrophy regression post aortic valve replacement – echocardiographic and cardiac MRI studies.

Author/year of publication	Imaging modality	Sample size	Inclusion criteria	Valve type	Mean follow-up	LVH regression (%LVMi change)
Repossini et al., 2012[118]	Echo	104	Good acoustic windows Normal LVEF	TAVR -Freedom solo bioprosthesis (stentless)	382 ± 163 days	1–3 months: −17.6% 12 months: −21.5% P < 0.05
Gotzmann et al., 2012[92]	Echo	202 Group 1: preserved LVEF, high gradient (N = 86) Group 2: preserved LVEF, low gradient (N = 27) Group 3: reduced LVEF, high gradient (N = 45) Group 4: reduced LVEF, low gradient (N = 44)	-Severe symptomatic AS -High risk for SAVR -Aortic annular diameter 20–27 mm and ascending aorta diameter <45 mm -Age ≥ 75 years with EuroSCORE ≥ 15% OR age > 60 with ≥ 1 specified risk factor	TAVR -Medtronic CoreValve Percutaneous System	12 months	Group 1: −9% Group 2: −13.5% Group 3: −21.1% Group 4: −21.7% P = 0.008
Jin et al., 1996[117]	Echo	137	- Age > 55 years -Single AVR ± CABG -Willingness to attend post-operative echo follow-up -Echocardiographic records technically adequate for computer digitizing	Aortic homograft (N = 39) Toronto stentless porcine valve (N = 72) Stented porcine or bileaflet mechanical valve (N = 26)	12 months	0.5 months: −25% 6 months: −39% 12 months: −36% 24 months: −36% 36 months: −39% P < 0.0001
Breitenbach et al., 2012[120]	Echo CMR	149	-Legal age in host country -First AVR for severe AS or mixed AV disease with predominant AS ± CABG	SAVR -Stented porcine bioprosthesis (Epic or Epic Supra)	7.1 months	TTE: Epic: −31% Epic Supra: −25% CMR: Epic: −24% Epic Supra: −24% P < 0.0001
Sadaba et al., 2012[119]	Echo CMR	149	-Isolated AVR to treat AS or mixed AV disease with predominant AS and mild AR Exclusion: -Previous surgical valve replacement or repair -Previous stent procedure -Concomitant cardiac procedure other than ascending aorta replacement and CABG -Unstable angina -NYHA class IV -Significant ventricular wall motion abnormalities -Active endocarditis -Acute aortic dissection -Persistent or permanent AF -Hemodialysis -Severe claustrophobia -Pregnancy -Presence of implants precluding MRI	SAVR - Epic (N = 77) - Epic Supra porcine bioprosthesis (N = 72)	6 months	CMR: Epic: −31% Epic Supra: −25% P < 0.0001
Lamb et al., 2002[121]	CMR	29 -Predominant AS without significant AR (N = 12) -Predominant AR (N = 7) -Healthy controls (N = 10)	-Severe AS or AR -No significant coronary artery disease	SAVR	9 months	Post AVR for AS: −38.8% Post AVR for AR: −27.4% P < 0.05 pre vs post surgery
Beach et al., 2014[89]	Echo	4264	-Severe AS (defined by AVA <1cm^2^) ± CABG Exclusion: -Predominant AR -IE -Rheumatic valve disease -Indication for AVR other than AS	SAVR - Bioprosthetic valve	6.1 ± 4.0 years	2 years: −16% 10 years: −13%
Lim et al., 2008[122]	Echo	289 -AS (N = 177) -AR (N = 56) -Mixed AV disease (N = 56)	All referrals for AVR	SAVR -Homograft (N = 141) -Stentless (N = 148)	Median: 4.0 years	1 year: −10%

Echocardiography remains the first-line assessment tool, providing information on valve structure; valve hemodynamics, using Doppler-derived measurements; ventricular geometry using LV mass calculations; and numerous parameters of ventricular function including LVEF, strain, and diastology [91,92]. Following SAVR or TAVI, LV mass (LVM) regresses fastest in the first 6 to 12 months – achieving 20–30% LVM reduction at 1 year, with different temporal patterns depending on burden of comorbidities, vascular stiffness and hemodynamic performance of the prosthesis type [89,91,93–95].

Diastolic dysfunction improves later (~3 years) with further regression of LVH out to 10 years dependent on baseline hypertrophy and co-existent arterial hypertension with other factors likely to play a role as well (initial gradients, subsequent valve type, patient prosthetic mismatch, degree of post procedure aortic regurgitation) [96,97]. Baseline global longitudinal strain has been shown to be the strongest predictor of LVH regression in a cohort of severe AS patients post SAVR [98]. Multi-modality imaging predictors of reverse remodeling have been described in more detail elsewhere [99]. As discussed previously, CMR offers additional information about focal scar and changes in the cell and interstitial components of the LV mass (via ECV) [77]. Focal scar appears to be fixed at 9- and 12-months post SAVR, with de-novo LGE occurring in 5% to 18% of patients though peri-procedural myocardial vulnerability is poorly understood [77,80,100]. In contrast, interstitial and cell volumes regress by 15–20% at 12 months post AVR, with the reduction in cell volume greater than interstitial volume reduction resulting in an overall increase in ECV% (because of the change in ratio) [77]. More data on the temporal relationship of these changes, their associations and whether this can be therapeutically influenced requires further studies.

## 4. Arrhythmia post AVR

Conduction abnormalities commonly complicate AVRs and are caused by direct mechanical insult from valve implantation to the intrinsic conduction system [22,101]. In patients with severe AS at moderately increased operative risk there was a higher incidence of conduction disturbances requiring permanent pacemaker insertion in the patients undergoing TAVI [102]. A recent implantable cardiac monitor (ICM) study in AS patients with new left bundle branch block post-TAVI demonstrated a 61% rate of first arrhythmic episodes at 24 months. These comprised predominantly bradyarrhythmic episodes, followed by atrial fibrillation (AF) or flutter, and new-onset ventricular tachycardia (VT), affecting 35%, 28% and 21% of patients respectively [103]. AF also increases the risk of stroke and HF progression. ICMs detect arrhythmias with high sensitivity and specificity for up to 3 years, and allow determination of the terminal rhythm. ICMs also record heart rate variability (reduced in autonomic dysfunction) and physical activity (reduced in HF) [104]. Ventricular arrhythmia data post-AVR is very limited despite links with SCD, but there are well-established links between myocardial scar detected by LGE imaging, non-sustained ventricular tachycardia (NSVT; ≥3 consecutive ventricular beats, rate ≥120 bpm) and SCD in dilated and hypertrophic cardiomyopathy [105]. A previous 24 h monitor study reported a 4.8% rate of NSVT at 1-month and 2.1% at 1-year post TAVI [106]. Further studies are required to investigate arrhythmia as a cause of cardiac decompensation and cardiovascular mortality post AVR. Current work by our group is investigating the link between myocardial scar and cardiac arrhythmia in patients post AVR using ICMs (MASTER Study; NCT04627987).

Beta-blockers are commonly used to control tachyarrhythmias and other common indications such as previous myocardial infarction or heart failure. There is paucity of evidence on the association between beta blockade and long-term outcome following AVR. Data from the SWEDEHEART registry demonstrated an increased all-cause mortality risk associated with beta-blocker use in patients post AVR, which persisted upon post subgroup analysis of patients with AF [107]. While a commonly prescribed drug for common cardiovascular conditions, safety and efficacy of beta-blocker use in AS patients following AVR merits further investigation.

## 5. Management of heart failure post AVR

**Figure 2.**
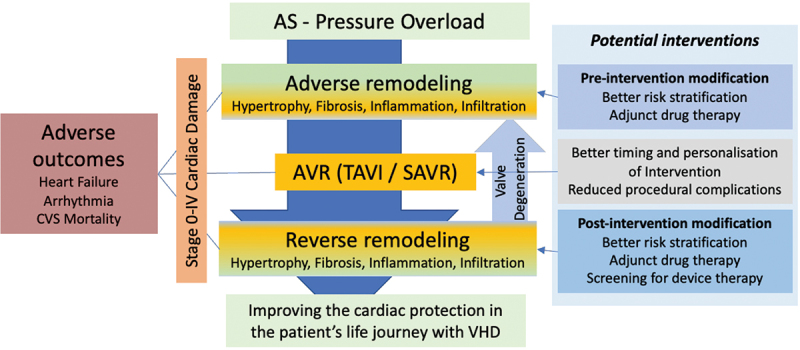
Potential strategies to mitigate excess morbidity and mortality associated with aortic stenosis. Optimal timing and tailoring of valve intervention to the patient with aortic valve replacement, with appropriate pre- and post-intervention modifications to treatment, may improve outcomes for patients with AS. *AS = aortic stenosis, AVR = aortic valve replacement, TAVI = transcatheter aortic valve replacement, SAVR = surgical aortic valve replacement, VHD = valvular heart disease.*

Outcomes following AVR remain a key focus of study and may inform management strategies to enhance these, such as for post-operative arrhythmias and heart failure (Table 2). Medical therapeutic options for management of the aortic valve stenosis itself have failed to emerge despite decades of randomized trials [108]. In contrast, myocardial remodeling and AS-associated myocardial damage could be attenuated with pharmacotherapy prior to or after AVR (Figure 2). Targets for intervention range from myocardial hypertrophy to inflammation and diffuse myocardial fibrosis, with better understanding of the factors and pathways driving heart failure post AVR required. Whereas there is a wealth of evidence for drug intervention targeting these pathways, and novel medical therapies currently emerging, there is limited (if any) trial data in patients with severe valvular heart disease before or after intervention, as this patient group was most excluded from previous trials [109].

**Table 2.** Studies of post-operative heart failure/arrhythmia/outcome.

Author/Year	Study type	Sample size	Treatment arm 1	Treatment arm 2	Follow-up	Primary endpoints	Secondary endpoints	Main results
UK TAVI Trial Investigators, 2022 [102]	RCT	912	TAVR (N = 458)	SAVR (N = 455)	Minimum 5 years, ongoing	All-cause mortality at 1 year	-Cardiovascular death -Stroke -Reintervention -Composite of death or stroke -Composite of death or disabling stroke -Composite of death, disabling stroke or reintervention -Vascular complications -Major bleeding events -Conduction disturbance requiring permanent pacing -Myocardial infarction -Kidney replacement therapy -Infective endocarditis	Primary endpoint All-cause mortality 4.6% vs 6.6%, p = 0.23 Secondary endpoints Cardiovascular death 2.8% vs 3.3%, p = 0.69 Stroke 5.2% vs 2.6%, p = 0.07 Major bleeding events 7.2% vs 20.2%, p < 0.001 Conduction disturbance requiring permanent pacing 14.2% vs 7.3%, p < 0.001 Vascular complications 10.3% vs 2.4%, p < 0.001
Muntane-Carol et al., 2021 (MARE investigators) [103]	Prospective multicentre study	103	TAVR -Sapien XT/3 (N = 53)	TAVR -CoreValve/EvolutR (N = 50)	2 years	-Incidence of arrhythmic events leading to treatment change -Incidence of adjudicated HAVB	N/A	Primary endpoints Arrhythmic events requiring treatment 18% vs 20%, p = 0.83 HAVB: 3% vs 0, no P value Pacemaker implantation 4% vs 7%, p = 1.00 Cumulative rate of first arrhythmic event at 2-year follow-up Global arrhythmic burden: 61% HAVB: 16% Pacemaker implantation: 15%
Gilard M. et al, 2012 (FRANCE 2 investigators) [30]	Prospective multicentre study	3195	TAVR -Edwards SAPIEN (N = 2107)	TAVR -Medtronic CoreValve (N = 1043)	Median follow-up 114 days	All-cause mortality	Safety: -MACCE -Cardiac events -Cardiac or vascular surgery -Bleeding -Stroke -NYHA functional class Efficacy: -Procedural success	Primary endpoints All-cause mortality (1 year) -SAPIEN vs CoreValve: 24% vs 23.7% -TF vs TA vs subclavian approach: 21.7% vs 32.3% vs 25.1% (p < 0.001) Secondary endpoints Stroke -Valve: 1.9% vs 2.6% -Approach: 2.2% vs 2.1% vs 2.7% P = 0.88 MI -Valve 0.8% vs 1.9% -Approach: 0.8% vs 1.8% vs 3.3% P = 0.004 Major bleeding -Valve: 2.0% vs 1.5% -Approach:1.5% vs 3.4% vs 3.3% P < 0.001 Efficacy endpoint Procedural success -Valve: 97.0% vs 97.6% -Approach: 97.1% vs 95.9% vs 96.7% P = 0.35
Banovic M. et al., 2022 (AVATAR investigators) [25]	RCT	157	Early SAVR	Conservative	Median follow-up 32 months	Composite of all-cause mortality or MACE (composite of acute myocardial infarction, stroke, unplanned HF hospitalization needing intravenous diuresis or inotropes)	-In-hospital and 30-day post-operative mortality in operated patients in both groups -Repeat aortic valve surgery in operated patients in both groups -Repeated MACEs -Major bleeding -Thromboembolic complications -Time to death -Time to first hospitalization	Primary endpoint Composite of all-cause mortality or MACE 15.22% vs 34.7%, p = 0.02 No significant differences in secondary endpoints.
Van Mieghem N.M. et al., 2022 (SURTAVI investigators) [123]	RCT	1660	TAVR (N = 684)	SAVR (N = 796)	5 years	Composite of all-cause mortality or disabling stroke	-All-cause mortality -Cardiovascular mortality -Myocardial infarction -Stroke -Aortic valve-related reintervention and rehospitalizations -Prosthetic valve endocarditis and clinical thrombosis -Conduction disturbances requiring permanent pacemaker implantation	Primary endpoint All-cause mortality or disabling stroke 31.3% vs 30.8%, P = 0.85 Secondary endpoints Permanent pacemaker implantation: 39.1% vs 15.1%, P < 0.001 No significant difference in other secondary endpoints.
Smith CR et al., 2011 (PARTNER investigators) [124]	RCT	699	TAVR (N = 348)	SAVR (N = 351)	Median follow-up 1.4 years	Rate of death of any cause at 1 year in intention-to-treat population	-Cardiovascular death -NYHA functional class -Repeat hospitalization because of valve- or procedure-related clinical deterioration -Myocardial infarction -Stroke -AKI -Vascular complications -Bleeding -6-minute walk distance -Valve performance	Primary endpoint Death from any case at 1 year 24.2% vs 26.8%, P = 0.44 Secondary endpoints Vascular complications 18% vs 4.8%, P < 0.001 Major bleeding 14.7% vs 25.7%, P < 0.001 Stroke 8.3% vs 4.3%, P < 0.04 No significant difference in other secondary endpoints.
Makkar R.R. et al., 2020 (PARTNER 2 investigators) [38]	RCT	2032	TAVR -Sapien XT (N = 1011)	SAVR (N = 1021)	5 years	Composite of all-cause mortality and disabling stroke	−5 year incidence of death from any cause -Disabling stroke -Repeat hospitalization -AV valve re-intervention -NYHA functional class -Echocardiographic assessments	Primary endpoint Composite all-cause mortality and disabling stroke 5 years: 47.9% vs 43.4%, P = 0.21 Secondary endpoints 5 year all-cause mortality 46% vs 42.1% Hazard ratio 1.09 (0.95–1.25) Repeat hospitalization 33.3% vs 25.2% Hazard ratio 1.28 (1.07–1.53) AV re-intervention 3.2% vs 0.8% Hazard ratio 3.28 (1.32–8.13) Disabling stroke 9.8% vs 8.6% Hazard ratio 1.05 (0.77–1.44)
Leon M.B. et al., 2021 (PARTNER 3 investigators) [22]	RCT	950	TAVR -Sapien 3 (N = 496)	SAVR (N = 4545)	2 years	Composite of all-cause mortality, all stroke, and all cardiovascular hospitalization at 1 year	-Acute myocardial infarction -New onset atrial fibrillation -New pacemaker -New left bundle branch block -Coronary obstruction -Aortic valve re-intervention -Aortic valve infective endocarditis -Valve thrombosis	Primary endpoint Composite all-cause mortality, stroke and rehospitalisation 11.5% vs 17.4%, P = 0.007 Secondary endpoints New onset AF 7.9% vs 41.8%, P < 0.001 New left bundle branch block 20.8% vs 9.7%, P < 0.001 No significant difference in other secondary endpoints.
Kang DH et al., 2020 (RECOVERY investigators) [19]	RCT	145	Early SAVR (N = 73)	Conservative (N = 72)	Median follow-up 6.2 years	Composite of operative mortality or death from cardiovascular causes	-All-cause mortality -Repeat aortic valve surgery -Clinical thromboembolic events -Heart failure hospitalization	Primary endpoint Composite of operative mortality or death from cardiovascular causes 15% vs 1% P = 0.003 Hazard ratio 0.09 (0.01–0.67) Secondary endpoints All-cause mortality 21% vs 7% Hazard ratio 0.33 (0.12–0.90) Clinical thromboembolic event 6% vs 1% Hazard ratio 0.30 (0.04–2.31) Repeat aortic valve surgery 3% vs 0 Hazard ratio 0.19 (0.10–8.00) Heart failure hospitalization 11% vs 0 Hazard ratio 0.05 (0.00–1.05)
Saito S. et al., 2021 (REPRISE Japan investigators) [125]	Prospective multicentre single-arm trial	82	Transfemoral TAVR -LOTUS (N = 40)	Transaortic TAVR -LOTUS(N = 10) 21 mm substudy via TF approach (N = 15)	6 months	Primary safety endpoint: Composite of all-cause mortality, stroke, life-threatening and major bleeding events, stage 2 or 3 acute kidney injury, or major vascular complications Primary efficacy endpoint: Composite of all-cause mortality, disabling stroke, moderate or greater paravavlular regurgitation	-Moderate or greater paravalvular aortic regurgitation Other outcomes: -New-onset of AF or A.flutter -New permanent pacemaker implantation -Hospitalization for valve-related symptoms or worsening CHF	Primary safety endpoint 15% vs 60% vs 26.7% Primary efficacy endpoint 5.3% vs 10.0% vs 7.1% Results of statistical testing not reported.

Activation of the renin-angiotensin-aldosterone system (RAAS) is implicated in the myocardial remodeling in AS; raised myocardial angiotensin converting enzyme (ACE) concentrations are associated with AS and AR, which in turn is associated with increased collagen and fibronectin synthesis, leading to myocardial fibrosis [110]. Drugs inhibiting RAAS may thereby modulate these effects. Investigators of the RIAS trial demonstrated modest regression in LV mass following one year of ramipril use at incremental doses versus placebo in patients with moderate-severe AS [111]. Similar effects of RAAS inhibition with candesartan have been demonstrated post AVR by Dahl et al.; this was associated with LV mass regression in addition to improvement in LV S’, suggesting reverse LV remodeling was associated with improved LV systolic function [112]. But these studies were not designed or powered against cardiovascular mortality or heart failure hospitalization. Looking at potential future strategies, the antifibrotic effect of spironolactone is currently being investigated by a German consortium in patients undergoing TAVI [113].

Other strategies may include the anti-fibrotic effect of torasemide affecting the extracellular collagen processing, sodium–glucose cotransporter 2 inhibitors via their metabolic pathway effect, TGFβ1 signaling inhibitors like pirfenidone, and immunomodulators via reduction of myocardial inflammation [114]. Beyond myocardial remodeling, residual pulmonary hypertension and increased systemic vascular load drive poor outcomes after AVR, and are associated with worse outcomes after valve procedures. Targeted medical therapies may improve residual symptoms, quality of life and outcomes. Dedicated trials directed at post-operative AS patients are urgently needed to improve symptoms, quality and quantity of life.

## 6. Conclusions

Aortic valve stenosis causes adverse cardiac remodeling that is only partially reversed by AVR. Multi-modality imaging plays an essential role in not only diagnosing the valvular stenosis but also the myocardial and cardiac damage resulting from AS. Myocardial fibrosis and scarring is associated with excess morbidity, hospitalization for heart failure and mortality after AVR. Heart failure management is currently not tailored to patients with valvular heart disease after valve intervention, and further trials of drug therapies are required to prove efficacy in this patient population.

## 7. Expert opinion

Aortic stenosis has implications for patient outcomes, with even moderate aortic stenosis being associated with excess mortality [115]. Therefore, it is pivotal to diagnose AS at the right time, assessing its severity properly and instigating the appropriate management (watchful waiting or intervention). Echocardiography is the main diagnostic modality with CMR and CT offering additional anatomical, functional or tissue characterization biomarkers. Such management is reliant on appropriate infrastructure being in place for screening, surveillance and timely management, but this has been shown to have significant geographic and socioeconomic variation [116].

Intervening at the right time (and implanting the right valve) is essential to balance upfront risk of intervention and the lifetime risk of a prosthetic valve against progressive cardiac damage due to AS. Multiple randomized clinical trials are trying to define this ‘sweet spot’ for intervention. But valve intervention is not a total cure, and no matter how well we screen or surveil, there will always be some patients who present late – we therefore need therapies beyond AVR.

We do not know why people die after AVR, but death rates are high (22% mortality at 3.5 years; compared with an estimated population mortality rate of ~7% [QRISK3]). Without understanding the most prevalent mode of death (heart failure, arrhythmia or other), knowing where to direct research efforts into potential therapeutic strategies (drug or device-based) is challenging. Furthermore, conventional heart failure therapy (drug or device-based) relies on LVEF cutoffs, which are poor measures of LV systolic performance in AS before and after AVR when hypertrophy is present and LVEF is supra-normal, like in hypertrophic cardiomyopathy; ‘AS cardiomyopathy’ is essentially a HFpEF phenotype. Currently after successful SAVR or TAVI, AS patients are typically discharged without assessment or active management of residual risk; current guidelines offer no specific guidance on assessing risk post-AVR, particularly detecting and managing heart failure in VHD, with the exception of anticoagulation for atrial fibrillation and pacemaker implantation for high-grade atrio-ventricular block.

So how may peri-AVR valve care look differently in 5–10 years’ time (Figure 2)? With better understanding of the pathophysiological mechanism of myocardial damage, patients with AS may be risk stratified more precisely in their journey from mild to severe AS, obtain adjunct ‘cardio-protective’ therapies prior to AVR and their intervention may be more personalized with regard to timing and type of valve. Following intervention, repeated risk stratification may identify those who benefit from further adjunct pharmaco- or device therapies. Current management is focussed on a singular valve intervention, but many patients will require multiple valve interventions. The management of AS is a life journey that needs to not only focus on implanting the right prosthesis at the right time, but also optimizing patients medically all along this journey. Devising such personalized management plans should improve patients’ quality of life, morbidity and mortality. Patient-centered research initiatives are required for such a vision [109].

## Article highlights

- Aortic valve stenosis causes adverse cardiac remodelling that is only partially reversed by aortic valve replacement (AVR).
- Multi-modality imaging plays an essential role in evaluating valvular stenosis and the myocardial response to AS.
- Myocardial scarring is associated with excess morbidity and mortality after AVR.
- Heart failure management is currently not tailored to patients with valvular heart disease after valve intervention due to insufficient evidence to guide therapy in this patient group.
